# End-of-Life Utilization Among Decedents With Fragility Fractures: The Role of Dementia and Family Availability

**DOI:** 10.1093/geroni/igaf122.030

**Published:** 2025-12-31

**Authors:** Amy Cizik, Eli Iacob, Djin Tay, Katherine Ornstein, Caroline Stephens

**Affiliations:** University of Utah, Salt Lake City, Utah, United States; University of Utah, Salt Lake City, Utah, United States; University of Utah, Salt Lake City, Utah, United States; Johns Hopkins University, Baltimore, Maryland, United States; University of Utah, Salt Lake City, Utah, United States

## Abstract

Fragility fractures (FFx) are associated with high morbidity and mortality, particularly among persons with dementia. While families play a critical role in caring for these individuals, little is known about how comorbid dementia and family availability impact their end-of-life (EOL) outcomes. This retrospective population-based cohort study examined rates of EOL care utilization (emergency room, hospital, outpatient) in the last two years of life for 20,988 decedents with a history of non-metastatic FFx, focusing on the impact of dementia and family availability. Most decedents with FFx were female (65.0%) and non-Hispanic White (94.6%), with 35.8% having dementia, 82.8% having at least one living family member, and 45.6% having sustained a hip, pelvis, or femur fracture. Mean age at death was 81.7 (SD = 11.7). Adjusted regression models showed that comorbid dementia was associated with a 10% decrease in hospitalization rates (0.899 [0.874,0.924]), a 42% decrease in outpatient visits (0.583 [0.530,0.642]), but 13% increase in emergency visits (1.134 [1.083, 1.188]), compared to no dementia. However, compared to those without family, presence of a spouse with or without child(ren) was associated with a 23% and 35% increase in outpatient visits, respectively (1.230 [1.098,1.378]); (1.351 [1.181,1.547]). Healthcare providers should consider both the presence of dementia and family availability when planning EOL care for persons with FFx. Increased use of emergency services by those with comorbid dementia suggests alternative care pathways, such as use of palliative care or more frequent home-based services, may be needed to address the increased need for emergency medical attention near EOL.

